# Comparison of interferometric light microscopy with nanoparticle tracking analysis for the study of extracellular vesicles and bacteriophages

**DOI:** 10.1002/jex2.75

**Published:** 2023-02-23

**Authors:** Romain Sausset, Zuzana Krupova, Eric Guédon, Sandrine Peron, Alice Grangier, Marie‐Agnès Petit, Luisa De Sordi, Marianne De Paepe

**Affiliations:** ^1^ Micalis Institute, INRAE, AgroParisTech Université Paris‐Saclay Jouy‐en‐Josas France; ^2^ Myriade 68 boulevard de Port Royal Paris France; ^3^ Excilone, Departement R&D 6 rue Blaise Pascal, Parc Euclide, Bat. A Elancourt France; ^4^ STLO, INRAE Institut Agro Rennes France; ^5^ Laboratoire MSC Matière et Systèmes Complexes CNRS UMR 7057 Université Paris Cité Paris France; ^6^ Centre de Recherche Saint Antoine Sorbonne Université, INSERM Paris France

**Keywords:** bacterial vesicles, bacteriophages, extracellular vesicles, interferometric light microscopy, nanoparticle tracking analysis

## Abstract

Research on extracellular vesicles (EVs) and bacteriophages (phages) has been steadily expanding over the past decades as many of their roles in medicine, biology, and ecosystems have been unveiled. Such interest has brought about the need for new tools to quantify and determine the sizes of these biological nanoparticles. A new device based on interferometric light microscopy (ILM), the Videodrop, was recently developed for this purpose. Here, we compared this new device to two nanoparticle tracking analysis (NTA) devices, the NanoSight and the ZetaView, for the analysis of EVs and phages. We used EVs isolated from bacteria, fecal samples, bovine milk and human cells, and phages of various sizes and shape, ranging from 30 to 120 nm of diameter. While NTA instruments correctly enumerated most phages, the Videodrop detected only the largest one, indicating a lower sensitivity threshold compared to the NTA devices. Nevertheless, the performance of the Videodrop compared favourably to that of the NTA devices for the determination of the concentration of eukaryotic EV samples. The NanoSight instrument provided the most precise size distributions but the Videodrop was by far the most time‐saving device, making it worthy of consideration for studies conducted on a large number of samples composed of nanoparticles larger than 90 nm.

## 1 INTRODUCTION

The discovery of the importance of extracellular vesicles (EVs) in basic research, medicine and industrial applications has placed them in the limelight in the last decades. EVs are produced and released into the environment by eukaryotic and prokaryotic cells, for which they are important actors in intra‐ and inter‐species cell–cell communication. Human EVs are present in numerous bodily fluids, where they participate in numerous homeostatic processes, such as cellular proliferation, and, therefore, in human health and disease (Reviewed in Shah et al., 2018). EVs constitute a promising source of biomarkers for many diseases, including cancer and chronic cardiovascular diseases (Lane et al., 2018; Martin‐Ventura et al., 2022). Bacterial EVs also play important roles, notably in pathogenesis, as certain bacteria deliver toxic compounds and virulence factors through EVs during infection (Bitto et al., 2017; Bomberger et al., 2009; Codemo et al., 2018; Furuta et al., 2009; Sahr et al., 2022; Tartaglia et al., 2018). Bacterial EVs have also been found to be abundant in aquatic ecosystems (Biller et al., 2014). Finally, EVs have attracted enormous interest in medicine and the pharmaceutical industry as potential vaccines or vectors for the delivery of active therapeutical compounds, as well as in regenerative medicine (reviewed in Meng et al., 2020) and (Avalos & Forsthoefel, 2022).

The growth in the number of studies and applications involving EVs has driven the development of tools for the characterization of their size and concentration. Such knowledge is important not only for the standardization of studies and procedures (Thery et al., 2018), but also for the use of EVs as biomarkers, as, for example, the concentration and size of circulating EVs has been associated with several cardiovascular diseases (Shah et al., 2018). However, there are still no simple, rapid and reliable tools to determine the size distribution and concentrations of entire populations of EVs of various size. The large heterogeneity of EV size in most samples, which typically ranges from 20 to 200 nm, even for EVs from a single cell type, requires single‐particle measurements to obtain reliable values. Accurate enumeration of EVs by traditional epifluorescence microscopy (EPI) following staining with non‐specific dyes (i.e., dyes that would stain all EVs) is difficult and scarcely used, probably due to aggregation of lipophilic dyes. In addition, EPI does not provide information on the size of objects. On the contrary, transmission electronic microscopy (TEM) can be used to visualize EVs and estimate their approximate size, despite possible underestimation due to shrinkage (Chernyshev et al., 2015; Kotrbova et al., 2019), but is very challenging to use for enumeration.

Therefore, more recent techniques have been developed to determine the concentration and size of nanoparticles, such as tunable resistive pulse sensing (TRPS), nanoparticle flow cytometry (NFCM), dynamic light scattering (DLS), and nanoparticle‐tracking analysis (NTA), but these techniques all have certain limitations. TRPS relies on changes in impedance of a nanopore caused by the passage of a nanoparticle in an electrolyte fluid. Although highly valuable and accurate for the characterization of EVs that are relatively homogenous in size (Doyle & Wang, 2019; Maas et al., 2014), the dimension of the pores has to be adapted to the size of the analysed particles, which is not possible for very polydisperse samples (van der Pol et al., 2014). NFCM is a flow cytometry‐based technique that has been improved for the detection of nanoparticles (Lippe, 2018; Rossi et al., 2015; Zamora & Aguilar, 2018), but requires very long observation times or very expensive instruments. DLS is a bulk method that detects the temporal fluctuations of intensities of the light scattered by a population of nanoparticles following illumination by a laser (Doyle & Wang 2019). Although highly valuable for the study of very small particles, as for all bulk methods, it is prone to biases arising from sample heterogeneity (Filipe et al., 2010). NTA is, to date, the most widely used technique for the study of EVs. Like DLS and NFCM, NTA exploits the light scattered by nanoparticles upon illumination with a laser, but the trajectories of single nanoparticles are followed, making it possible to determine their hydrodynamic diameter (Dh), which is the diameter of a hard, perfect sphere with a zero surface charge that would diffuse at the same speed as the measured particle (for simplicity, we will use size to indicate Dh when discussing measurements provided by NTA). The ZetaView (Particle Metrix, Germany) and NanoSight (Malvern, UK) are two devices that rely on NTA commonly used in the EV field. Although they are based on the same principle, the ZetaView and NanoSight present important differences in the composition of their hardware and software (Bachurski et al., 2019). For example, with the NanoSight instrument, a flux is generally applied to the sample during acquisition, whereas the acquisition is recorded on a static sample with the ZetaView. In addition, ZetaView is capable of measuring particle motion under an applied electric field, which allows calculation of the zeta‐potential, a proxy for particle surface charge. On polystyrene and silica nanospheres, the ZetaView was shown to provide better concentration measurements, whereas the NanoSight provided greater precision in size estimations (Bachurski et al., 2019).

Despite the strengths of NTA, its main limitations result from the sixth‐power dependence of the scattered light intensity on the size of the particle, resulting in inaccurate measurements in polydisperse samples (Dehghani et al., 2021; Gardiner et al., 2013), and a threshold of detection of around 60 nm, which makes it unsuitable for the study of smaller vesicles (Bachurski et al., 2019). In addition, the use of NTA‐based devices is relatively time‐consuming, which is a practical limitation for studies that require a large number of samples. Therefore, there is still a need for a tool that is both very rapid (i.e., acquisition of results in few minutes) and appropriate for highly polydisperse samples. These properties are the advertised strengths of a new instrument based on interferometric light microscopy (ILM), the Videodrop (Myriade, France). As NTA, ILM uses Brownian motion to calculate the size distribution of the analysed particles. However, contrary to NTA, a simple LED illuminates the samples and a transmission bright‐field microscope is used as a homodyne interferometer to detect the particles due to the interference created by the superposition of the incoming light field and the light scattered by the nanoparticles. Being mostly in the Rayleigh scattering regime, the intensity of the scattered amplitude is proportional to the third‐power of the particle diameter (Hulst, 1957) and not to the sixth‐power, as in NTA, limiting the decrease of signal intensity with particle size.

The Videodrop was developed from an interferometer (Boccara et al., 2016; Roose‐Amsaleg et al., 2017), which has been used to enumerate nanoparticles in cheeses (Dugat‐Bony et al., 2020) and aquatic environments (Boccara et al., 2016; Roose‐Amsaleg et al., 2017). A recent study showed the Videodrop to correctly enumerate two lentiviral virions and a baculovirus, whereas it underestimated the absolute viral concentration of an adenovirus relative to classical titration procedures (Turkki et al., 2021). A Videodrop instrument was also used to estimate the concentration of EVs in plasma, but no control experiments to verify the performance of the device were done in this study (Sabbagh et al., 2021). The main characteristics of the Videodrop, ZetaView, and NanoSight instruments are summarized in Table 1.

**TABLE 1:** General characteristics of the Nanosight, the ZetaView, and the Videodrop devices. Information was collated using manufacturer websites.

	Videodrop	ZetaView	NanoSight
Technology	ILM	NTA	NTA
Light source	LED	Laser	Laser
Sample introduction	Droplet in a tank	Syringe, tubings	Syringe, tubings
Acquisition time	20 sec	1 min	1 min
Temperature control	No	Yes	Yes
Possibility of Fluorescence	No	Yes	Yes
Cost of consumables	∼ 0	$	$
Cost of equipment	$	$$	$$

In this study, we compared the performance of the Videodrop in determining the size and concentration of EVs with that of two NTA devices, the ZetaView and the NanoSight. To strengthen our analysis, we expanded our methodological comparison to biological nanoparticles of similar sizes but of different nature, bacteriophages (or simply phages). The phage particles represented benchmark comparisons, as they constitute monodisperse populations, the diameter of the capsid being highly homogenous among virions of the same species. Importantly, the refractive indices (on which depend the signal intensity) of viruses are relatively similar to those of EVs (between 1.42 and 1.49 for viruses vs. 1.36–1.39 for EVs) (Chandler et al., 2011; Gardiner et al., 2013; Holzwarth et al., 1974; Pang et al., 2016; van der Pol et al., 2012). In addition, in contrast to EVs, phages can be reliably quantified using various techniques, such as plaque counting, quantitative PCR and fluorescence microscopy (EPI). Furthermore, phage enumeration is of great interest in itself, since phages, as predators of bacteria, are important actors in all microbial ecosystems and, notably, in human‐associated microbiota (Sausset et al., 2020). Differences in phage composition have been shown, for example, to be associated with intestinal bowel diseases (IBDs), such as Crohn's disease and ulcerative colitis (Clooney et al., 2019; Cornuault et al., 2018; Norman et al., 2015). The rapid and reliable enumeration of phage particles is therefore of considerable interest, not only for understanding the dynamics of complex microbial ecosystems, but also for the development of phage‐based applications in biotechnology and medicine.

Here, we present the performance of the ILM and NTA devices for the observation of purified EVs and phages. We used nine types of EVs of very different origin, that is, originating either from milk, human or bacterial cells, or rodent faeces (germ‐free or raised conventionally), and purified using several procedures (differential centrifugation, iodixanol or sucrose density gradient and size exclusion chromatography). The phages were chosen to cover a large range of capsid diameters, from 30 to 120 nm, and to have different morphotypes: we used myophages and siphophages, which have long protein tails, and podophages and *Tectiviridae*, which have no or very short tails (Table 2). We also compared the size distributions obtained using the Videodrop and NTA devices to those obtained by transmission electron microscopy (TEM), knowing that the sizes derived from Brownian motion do not equate with the geometrical diameters given by TEM, and that the size of EVs can be reduced by 15%–30% during TEM observations (Chernyshev et al., 2015).

**TABLE 2:** Characteristics of the phages used in this study. Capsid diameters are derived from Viral Zone (viralzone.expasy.org), except for SPP1 (Ignatiou et al. 2019)).

	**T4**	**P1**	**T5**	**λ**	**SPP1**	**ΦcrAss001**	**T7**	**PRD1**	**ΦX174**
Family or morphotype	Myophage	Myophage	Siphophage	Siphophage	Siphophage	Podophage	Podophage	*Tectiviridae*	*Microviridae*
Capsid diameter (nm)	120 x 86	85	90	60	61	76	60	66	30
Genome type	dsDNA	dsDNA	dsDNA	dsDNA	dsDNA	dsDNA	dsDNA	dsDNA	ssDNA
Genome size (kb)	169	100	121	48	44	103	40	15	5

We show that the Videodrop is less sensitive than the NTA instruments, which makes it unsuitable for enumerating most phages, which are too small. However, the Videodrop estimates concentrations similar to those obtained by NTA for most EVs derived from mammalian cells. In addition, the Videodrop was by far the most time‐saving device, making it the most appropriate for studies conducted on a large number of samples comprised of sufficiently large objects.

## 2 MATERIALS AND METHODS

### 2.1 Phage lysates

*Escherichia coli* and *Bacillus subtilis* cultures were grown at 37°C in Luria broth (LB) supplemented with 5 mM MgSO_4_, 5 mM CaCl_2_, and 0.2% maltose for lambda phage. The cultures were infected during exponential growth (OD_600 nm_ of 0.2). *E. coli* phages were grown on the MG1655 *hsdRM* strain (MAC 1403, kanR) at a multiplicity of infection (MOI) between 0.1 and 0.25, except for PRD1, which was grown at 37°C on *E. coli* HMS174 pL2 (with kanamycin at a final concentration of 25 μg/mL), and ΦX174, which was grown at 30°C on *E. coli* C. For both a MOI of five was used. SPP1 was grown on *B. subtilis* YB886 using a MOI of 0.01. ΦCrAss001 was grown as described by Shkoporov et al. (2018). Briefly, *Bacteroides intestinalis* (DSM 108646) was grown in fastidious anaerobic broth (FAB) under anaerobic conditions and infected at a MOI of 1. All infections were performed in 500 mL exponentially growing bacterial cultures (OD_600 nm_ of 0.2) and incubated until deceleration of bacterial growth was observed. Bacteria and debris were then pelleted by centrifugation at 5200 × *g* for 30 min and the supernatants filtered using a vacuum‐driven Stericup filtration system at 0.22 μm (Merck Millipore).

### 2.2 Extracellular vesicle preparations

Bacterial culture supernatants containing EVs were obtained from 500‐mL cultures grown at 37°C. *Faecalibacterium prausnitzii* L2‐6 was grown in an anaerobic chamber filled with 5% H_2_, 5% CO_2_, and 90% N_2_ in sterile brain heart infusion supplemented (BHIS) medium supplemented with L‐cysteine (0.5 g/mL), maltose (1 g/mL), and cellobiose (1 g/mL) for 24 h. *Staphylococcus aureus* HG003 was grown in BHI medium and *B. subtilis* YB886 and *E. coli* Nissle 1917 in LB, all with 150 rpm/min agitation in a 1 L flask for 18 h. All cultures were centrifuged at 10,000 × *g* for 20 min and the supernatant filtered through 0.22‐μm Stericup Millipore filters. EVs from murine faeces and caecal content were collected either from 8‐week‐old C57BL/6NRj males (Janvier Labs) or 10‐week‐old germ‐free C3H/HeN and C57BL/6J mice from the Anaxem animal facility (INRAE, Jouy‐en‐Josas, France), both maintained in a 12 h‐light/12 h‐dark cycle and fed a chow diet (Ssniff). Rat faeces was collected from germ‐free F344 Fisher rats grown in the Anaxem facility. To obtain faecal filtrates, fresh or frozen (at −80°C, immediately after sampling) material was diluted 40‐fold in cold 10 mM Tris (pH 7.5) and resuspended by gentle agitation at 4°C for 15 min. After centrifugation at 5200 × *g* for 30 min, the supernatant was filtered using 0.22 μm pore‐size Pall Acrodisc syringe filters.

### 2.3 Purification of bacterial EVs and phages

Phage lysates and EVs from *B. subtilis* and *F. prausnitzii* supernatants, prepared as described above, were concentrated by centrifugation at 20,000 × *g* for 16 h (rotor SS‐34, Sorvall RC 5C PLUS). Pellets containing phage were resuspended in 0.5 mL SM buffer (200 mM NaCl, 50 mM Tris pH7, 10 mM MgSO_4_) and those containing EVs in Tris 10 mM pH7. Resuspended pellets were then subjected to an iodixanol density gradient in 5‐mL Ultra‐Clear centrifuge tubes (Beckman Coulter). The tubes were first filled with a two‐layer gradient, in which 1.875 mL 45% iodixanol in SM buffer (density 1.25) was gently injected under 2.5 mL 22% iodixanol in SM buffer (density 1.12), and the resuspended pellets then layered on the top. The tubes were ultracentrifugated at 100,000g × *g* for 5 h at 4°C (SW55Ti rotor in a XL‐90 Beckman Coulter centrifuge. One millilitre fractions containing a band at the expected density for EVs (around 1.11 g/mL, see Optiprep Application Sheet S60), or phages (1.18 g/mL, see Optiprep Application Sheet V38) were extracted from the top (Figure S1). Fractions of interest were dialyzed overnight at 4°C against 1 L of 10 mM Tris for EVs or SM buffer for phages, under agitation, using 25 kD Spectra/Por dialysis membranes. A second dialysis of 3 h was performed the next day under the same conditions. Cell‐free supernatants from *S. aureus* cultures were subjected to EV isolation and purification by sucrose density gradient ultracentrifugation, as described previously (Luz et al., 2021; Tartaglia et al., 2018). Cell‐free supernatants from *E. coli* cultures were subjected to EV isolation and purification by size‐exclusion chromatography, as described previously (Rodovalho et al., 2020). All bacterial EV samples were stored at 4°C and analysed within the following 2 weeks. At 4°C, concentrations and size distribution, as determined with the Videodrop, of phage and bacterial EV samples described above were shown to be stable for several months.

### 2.4 Enrichment of THP1 EVs

THP1 cells (ATCC) were cultivated in RPMI medium supplemented with 10% FBS and 1% penicillin‐streptomycin at 37°C in 5% CO_2_. They were maintained at a concentration of between 3 × 10^5^ and 1 × 10^6^ cells/mL. For EV production, the cells were washed with RPMI + 1% penicillin‐streptomycin without serum and then resuspended in the same medium in T175 flasks at a concentration of 2.5 × 10^5^ cells/mL in 50 mL. After 48 h, the conditioned media was harvested and centrifuged for 10 min at 2000 × *g*. The supernatant was ultracentrifuged for 2 h at 150,000 × *g* in an Optima XP centrifuge (Beckman) with an MLA‐50 rotor. The EV pellet was resuspended in 0.8 mL sterile PBS. The EVs were aliquoted and stored at −80°C until further analysis.

### 2.5 Isolation of bovine milk‐derived EVs

Whole bovine milk samples (200 mL) were centrifuged at 3000 × *g* for 15 min at 4°C (Allegra X‐15R, Beckman Coulter, France) to separate the fat from the skimmed milk. The whey was obtained after acid precipitation of the skimmed milk with 10% (v/v) acetic acid at 37°C for 10 min followed by the addition of 10% (v/v) 1 M sodium acetate and a further incubation of 10 min at room temperature. The precipitate was then centrifuged at 1500 × *g* at 4°C for 15 min. The supernatant was filtered using a vacuum‐driven 0.22‐μm filtration system Steritop (Merck Millipore). The whey supernatants were concentrated by centrifugation at 4000 × *g* at 20°C using Amicon 100‐kDa centrifugal filter units (Merck Millipore) to a final volume of ∼6 mL. Aliquots of 500 μL of the obtained retentate were loaded onto a qEVoriginal 70 nm SEC column (Izon Science, New Zealand) previously washed and equilibrated with PBS. Fraction collection (500 μL per fraction) was immediately carried out using PBS as the elution buffer. The selected elution fractions (1–3 of 500 μL each) were pooled and subsequently concentrated using 100‐kDa Amicon centrifugal filter units (Merck Millipore). The concentrated samples were subjected to several washing steps with PBS to obtain a highly pure EV population. The EV standards were aliquoted and stored at −80°C until further analysis.

### 2.6 Transmission electron microscopy (TEM)

Purified EVs or phage samples (10 μL) were directly adsorbed onto a carbon film membrane on a 300‐mesh copper grid, stained with 1% uranyl acetate dissolved in distilled water, and dried at room temperature. Grids were examined using a Hitachi HT7700 electron microscope operated at 80 kV (Elexience) and the images acquired with a charge coupled device camera (AMT). This work was carried out at, and with the expertise of, the MIMA2 platform, INRAE (Jouy‐en‐Josas, France).

### 2.7 Plaque assay (PA)

Ten microliters of the appropriate dilution of the purified phage preparations were mixed with 300 μL of a culture of their bacterial hosts grown overnight in the conditions used for phage lysates (see above). For all phages, except ΦcrAss001, the phage‐bacteria suspensions were mixed with 5 mL warm soft top agar (0.45% w/v agar, 0.25% w/v NaCl, 0.1% w/v Bacto Tryptone; in osmosis‐purified water) and immediately poured into Petri dishes already containing a solid LB agar layer (1.5% w/v agar and 2.5% w/v LB powder) in triplicate. For ΦcrAss001, the published protocol was followed (Shkoporov 2018). Briefly, the anaerobically prepared phage‐bacteria suspension was mixed in 5 mL 0.4% Bacto agar and immediately poured into three Petri dishes already containing a solid layer of fastidious anaerobic agar (FAA). After solidification, the Petri dishes were incubated overnight at 37°C, except for ΦX174 (30°C). The next morning, lysis plaques were manually counted and the phage titers in plaque‐forming units per millitre (PFU/mL) were calculated.

### 2.8 Quantitative PCR (qPCR)

DNA standards for calibration were prepared from phage genomic DNA as follow. Prior to genomic extraction, 500 μL of high‐titer phage samples were treated with 0.50 μL Turbo DNAse I (Ambion, 2 U/μL) and 1 μL RNAse A (10 mg/250 mL) at 37°C for 30 min. After adding EDTA to a final concentration of 10 mM to inactivate nucleases, phage DNA was extracted by two phenol‐chloroform‐isoamyl alcohol (25:24:1) extractions followed by three chloroform‐isoamyl alcohol (24:1) purification steps. DNA was precipitated with two volumes of ethanol and 300 mM potassium acetate pH 4.8 and resuspended in 10 mM Tris buffer pH 8. DNA was quantified using a Qubit® device and diluted in 10 mM Tris pH 8 to obtain a concentration 5.0 × 10^6^ genomes of phage in 6 μL. The sample was further diluted four times 3 fold to obtain the calibration range. Phage samples were quantified after treatment with Turbo DNAse I (1 μL for 1 mL of phage sample diluted 10 times in Tris 10 mM pH8) for 1 h at 37°C followed by 30 min at 95°C to explode capsids and degrade the DNase. The primers shown in Table S1 were used at a concentration of 10 μM. qPCR was performed in a total volume of 15 μL in MicroAmp Fast Optical 96‐well plates sealed with MicroAmp Optical Adhesive Film using the Takyon ROX SYBR Mastermix blue dTTP kit. Amplifications were run in duplicate on a StepOnePlus real‐time PCR system with the following cycling conditions: 95°C for 5 min (95°C for 15 s, 58°C for 45 s, 72°C for 30 s) for 45 cycles, 72°C for 5 min, 95°C for 15 s, 60°C for 15 s, 95°C for 15 s. All phage preparations were independently quantified three times. The analysis of the melting curves confirmed the specificity of the primers. Data analysis was performed using the manufacturer's StepOne Software 2.3.

### 2.9 Epifluorescence microscopy (EPI)

Phage samples were diluted to a concentration of approximately 10^7^ virions/mL. Glutaraldehyde was added to 1 mL of diluted sample to a final concentration of 0.5% and incubated at 4°C for 15 min. Samples were then flash frozen in liquid nitrogen. After thawing, 4 mL SM buffer was added to each sample prior to filtration on 0.02‐μm Anodisc filters. Each filter was then incubated on a 50‐μL drop of SYBR Gold at 200× in the dark for 15 min, with the virus side up. After removal from the drop, filters were dried in the dark before being mounted on a glass slide with Fluoromount‐G and a coverslip. Slides were stored at −20°C until observation. Microscopic observations were carried out using a Nikon Ti‐E fitted with a 100× oil objective Apo TIRF (NA, 1.49; Nikon) with an iLas2 laser coupling system from Gataca Systems (150 mW, 488 nm). Ten images were captured per slide in the bright field and GFP fluorescence channels (with an excitation filter wavelength of 472/30 nm and emission filter wavelength of 520/35 nm). Emission was collected using interference filters and the images captured using a pair of sCMOS cameras (Orca Flash 4.0 v2 sCMOS; Hamamatsu), with the gain defined at 300, attached to a ×2.5 magnification lens, with a time exposure of 100–200 ms, depending on the fluorescence intensity of the phage. The final pixel size was 64 nm. Metamorph v.7 software packages were used to control and process the image acquisition and the images were further analysed using ImageJ (v1.52a). The number of phages on the whole filters was calculated by multiplying the average counts by the quotient of the area of the filter in contact with the phages by the area of the images.

### 2.10 Polystyrene beads

For linearity measurements, polystyrene beads (Polystyrene Nanosphere Suspension Series 3000, ThermoFisher) of various sizes were diluted in water after sonication for 20 s. Bead concentrations were calculated following the indications of the Nanosphere supplier provided in the technical guide.

### 2.11 ILM measurements with the Videodrop

All purified samples were diluted to the appropriate concentrations (20–100 particles per frame) in their respective buffers and 6‐μL drops were used for the measurements. Measurements of the buffer alone were systematically performed. Triplicates of the accumulation for 10 acquisitions of 100 frames were recorded per sample in accumulation mode. In accordance with the manufacturer's specifications, a relative threshold of 3.8 was applied for detection. The removal of macroparticles was enabled using a minimum radius of 10 and a minimum number of hot pixels of 80, as well as drift compensation. Concerning the tracking settings, a maximum of two jumps was tolerated for a minimal length track of 10 frames. The doublet detector (qvir software version 2.5.2.6196) was used for all measurements. Size distributions were obtained after the application of a mobile mean with a period of three on histograms with classes of 5 nm.

### 2.12 NTA measurements with the PMX 220 ZetaView

The Zetaview system (Particle Metrix, Germany) was equipped with a 488‐nm laser. Measurement concentrations were obtained by pre‐diluting the samples to the ideal 50–200 particles/frame. Each experiment was performed in duplicate on 11 different positions within the sample cell with the following specifications and analysis parameters: cell temperature 25°C, sensitivity 70, shutter 100, Max Area 1000, Min Area 10, and Min Brightness 25. The results were validated while obtaining at least 1000 valid tracks for each run. For data capture and analysis, Nanoparticle Tracking Analysis Software (ZNTA) v 8.05.04 was used. Size distributions were obtained after the application of a mobile mean with a period of three on histograms with 5‐nm classes.

### 2.13 NTA measurements with the NanoSight NS300

All purified samples were diluted in their appropriate sterile buffer to a volume of 1 mL at an ideal concentration of 20–50 particles per frame and injected at a speed of 50 μL/s into the machine's specimen chamber with a 1‐mL sterile syringe. Measurements of the buffer were systematically performed. For each measurement, five acquisitions of 1 min were recorded at 25°C (except for polystyrene beads, for which only three acquisitions were recorded). The device was equipped with a sCMOS camera and a laser module of 488 nm for most experiments, except for polystyrene beads, phages PRD1 and ΦX174 and milk EVs, for which a laser module of 405 nm was used. Observations of T4 and PRD1 phages with both laser modules (405 and 488 nm) showed almost no differences in concentration or size. A camera level of 15 was used in all experiments except for polystyrene beads of 100, 150 and 200 nm, for which camera levels of 13, 11 and 8 were used, respectively. After capture, the videos were analysed using NanoSight software NTA 3.3 Dev Build 3.3.104, with a detection threshold of four. Size distributions were obtained after the application of a mobile mean with a period of three on histograms with 5‐nm classes using Excel.

## 3 RESULTS

### 3.1 Evaluation of the linearity range of the three instruments on polystyrene beads

First, we observed standardized polystyrene beads of known sizes and concentrations at various dilutions with the three instruments. In the case of the Videodrop, there was good agreement between the theoretical and measured concentrations between 5 × 10^8^ and 4 × 10^9^ beads/mL for beads with a diameter ≥80 nm (Figure S2a,b). Below 5 × 10^8^ beads per mL, concentrations are too close to the concentrations determined in buffer (3 × 10^8^ NPs/mL) to be accurate. The concentrations of 70‐nm beads were underestimated by 5 to 10‐fold, and 80‐nm beads by two‐fold, indicating that 80 nm is the Videodrop size threshold for the good enumeration of polystyrene beads. Bead sizes were properly estimated (error range below 5%) for beads comprised between 100 and 150 nm, while the size of 80 nm and 200 nm beads were a little bit less precisely estimated (error range around 10%–15% (Figure S2c).

By comparison, the concentrations obtained with the ZetaView were in good agreement with theoretical concentrations between 1.10^7^ and 2.10^8^ beads/mL for the 80‐nm, 100‐nm and 150‐nm beads (70‐nm and 125‐nm beads were not tested). However, the optimal concentration range was much narrower for 200‐nm beads (Figure S2d,e). Except for the 150 nm beads, concentrations measured were slightly above the theoretical concentrations. For all beads, the sizes were correctly estimated within a 10% error range (Figure S2f).

The NanoSight had the larger optimal concentration range, providing good agreement between theoretical and measured concentrations for 100‐nm diameter polystyrene beads at concentrations between 1 × 10^7^ and 1 × 10^9^ particles/mL. For all beads, the sizes were correctly estimated within a 10% error range (Figure S2i). However, in contrast to the other two instruments, acquisition parameters had to be adjusted to the bead size. Indeed, with the camera level adapted to 80‐nm beads, some 150 or 200‐nm beads create very intense signal, that completely fill the field of view and prevent good detection of other particles. In the rest of our study, we used the optimal parameters for 80‐nm beads, closer to our biological objects, but this raises questions about measures in highly polydisperse preparations containing both 80‐nm and 200‐nm objects.

### 3.2 Determination of phage concentration

**FIGURE 1:**
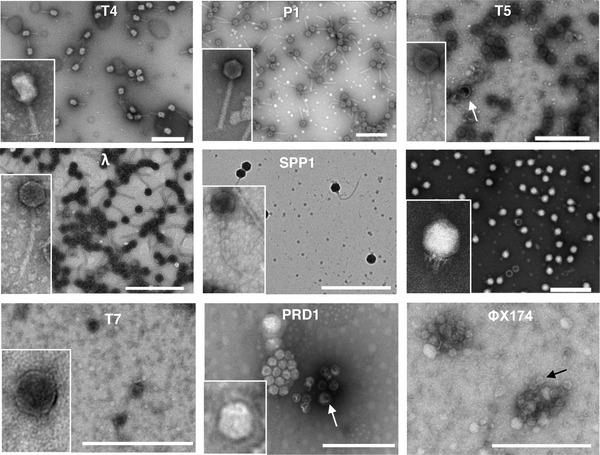
Representative TEM images of phage samples. White arrows point to EVs in PRD1 and T5 images. The black arrow in PhiX174 points to a virion. Scale bars are 500 nm.

We then evaluated the performance of the Videodrop and the two NTA devices for measuring the concentrations and sizes of our benchmark biological nanoparticles, that is, phages of known concentrations and sizes. Thus, we first purified phage virions of nine species belonging to different taxonomic families with different virion shapes and sizes (Table 2). TEM observations showed the phage preparations to be almost free of EVs and other large contaminants (Figure 1). When non‐phage objects were observed, as in the PRD1 and T5 preparations, they were more than 10‐fold less abundant than the phage particles. In the PRD1 and ΦX174 preparations, the virions appeared to be aggregated on the TEM images, but the subsequent size measurements obtained by NTA indicated that this aggregation occurred on the TEM grids. The capsid and tail dimensions measured for each phage were close to published values (Tables 2 and 3).

**TABLE 3:** Phage characteristics measured by TEM and NTA. SD : Standard deviation, Dh : Hydrodynamic diameter, nr: Not relevant, ‐: Not measurable. The mode is the value which correspond to the peak of the distribution, and the span = (D90 – D10)/D50, with D10, D50 and D90 being the sizes below which 10%, 50% or 90% of all particles are found respectively. The span gives an indication of how far the 10 percent and 90 percent points are apart, relative to the median.

		**T4**	**P1**	**T5**	**Lambda**	**SPP1**	**ΦcrAss001**	**T7**	**PRD1**	**ΦX174**
TEM	Capsid diameter mean ± SD (nm)	110 ± 6 × 85 ± 7	87 ± 5	79 ± 5	64 ± 3	58 ± 2	90 ± 4	64 ± 4	65 ± 4	26 ± 3
	Tail length mean ± SD (nm)	119 ± 9	235 ± 11	195 ± 18	153 ± 13	181 ± 21	25 ± 5	Very small	No tail	No tail
	Tail width mean ± SD (nm)	16 ± 2	18 ± 3	9 ± 2	9 ± 2	9 ± 2	Very small	Very small	No tail	No tail
NanoSight	Dh mode (nm)	158	168	128	88	65/95	94	64	71	–
	Dh span (nm)	0.26	0.30	0.33	0.59	nr	0.15	0.25	0.49	–
ZetaView	Dh mode (nm)	135	166	119	232	–	98	156	158	–
	Dh span (nm)	2.74	0.9	3.02	2.22	–	4.80	4.90	2.32	–

**FIGURE 2:**
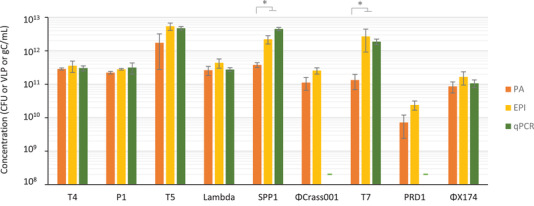
Phage counts by standard techniques. Phage concentrations were determined by Plaque Assay (PA, orange), Epifluorescence Microscopy (EPI, yellow), and quantitative PCR (qPCR, green). Error bars represent standard error of the mean on three independent experiments. Dashes (‐) indicate absence of measurement. * indicates Wilcoxon ‐ Mann Whitney test *p*‐value < 0.1.

The concentrations of phage preparations were determined using three reference methods: EPI, PA and qPCR. In cases where EPI and qPCR were realized on the same samples (seven out of the nine samples), the techniques provided comparable concentrations (not significantly different with a Wilcoxon‐Mann Whitney test, *p*‐values comprised between 1 and 0.2), comforting the reliability of the techniques (Figure 2). We thus used the concentrations obtained by EPI as the reference value for further comparisons. The concentrations obtained by PA were similar to or only slightly lower than those obtained by EPI for most phages (Wilcoxon‐Mann Whitney test, *p*‐values comprised between 1 and 0.2), but for phages T7 and SPP1, the differences were greater and significant (Wilcoxon‐Mann Whitney test, *p*‐values < 0.1). This is likely related to the presence of non‐infectious virions, unable to form plaques, as regularly reported (Heider & Metzner 2014; Huang & Baltimore 1970), in particular after thorough purification, which can damage the particles. In support of this hypothesis, we observed tailless phage particles by TEM in the purified SPP1 stocks (Figure 1).

**FIGURE 3:**
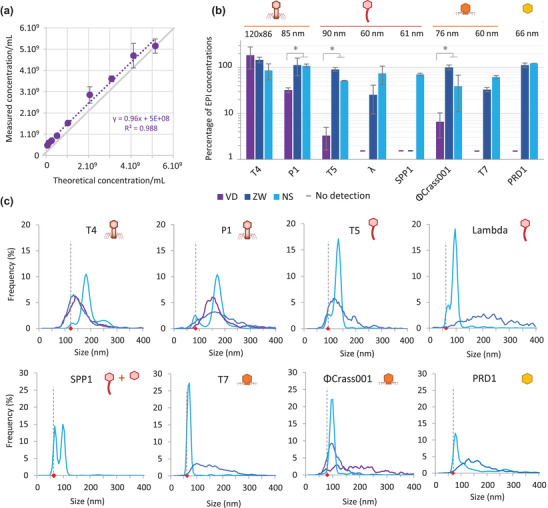
Comparison of ILM and NTA for the analysis of phages. (A) Measurements on serial dilutions of T4 with the Videodrop. (B) Relative phage concentrations compared to EPI values (% of the concentration determined by epifluorescence) obtained with the Videodrop (purple), the ZetaView (dark blue) and the NanoSight (clear blue). Error bars represent standard deviation on three independent experiments. Numbers above graph indicate phage capsid diameters in nm. Dashes (‐) indicate that the instrument provided no reliable measurement (<1% of EPI values). * indicates Wilcoxon ‐ Mann Whitney test *p*‐value < 0.1. (C) Virion hydrodynamic diameter (Size) distributions for each device. Colors are as in (B). Black dashed bars and red diamonds indicate the geometrical diameter of phage capsids.

We next compared the performance of the Videodrop and the two NTA devices in determining phage concentrations. First, we measured the concentrations of serial dilutions of T4 phage with the Videodrop (Figure 3a). The determined optimal concentration range matched the range determined previously with the polystyrene beads, that is, from 3 × 10^8^ to 5 × 10^9^ particles/mL. All further measurements with the Videodrop were therefore carried out in this concentration range. For the NanoSight and Zetaview, we used the optimal concentration range determined previously with polystyrene beads, that matched the optimal ranges recommended by the manufacturers.

The NanoSight detected all tested phages, except the smallest, ΦX174, for which the capsid diameter is only 30 nm. The NanoSight phage detection threshold was therefore between 30 and 60 nm. The concentrations obtained were in relatively good agreement with the EPI counts, with values that were generally 1‐to‐3‐fold lower for all phages (Figure 3b). The ZetaView also detected most of the phages except two: ΦX174, as the NanoSight, as well as SPP1. This is surprising, as the published diameter of the SPP1 virion, 61 nm, is similar to that of *λ* and T7 phages (Table 2), which were correctly detected. Of note, SPP1 particles create a visible signal that is nonetheless too low to be detected by the ZetaView software with the parameters used in this study, indicating that SPP1 virions are just below the detection threshold used. This may result either from a slightly smaller size of the SPP1 virions, as suggested by our TEM observations indicating a diameter of approximately 57 nm (Table 3), from a lower refractive index of SPP1 virions relative to other phages, or from a smaller surface charge (which results in a smaller hydrodynamic diameter). We therefore considered that the detection threshold of the ZetaView is slightly over 60 nm for phages. For phages with a capsid larger than 60 nm (T4, P1, T5, ΦCrAss001 and PRD1), the ZetaView concentrations were in very good agreement with the EPI counts (Figure 3b).

The Videodrop correctly detected only the largest phage, T4. P1 phage appeared to be well detected, but the concentration was underestimated by almost three‐fold, suggesting that a large proportion of particles were not correctly detected. T5 and ΦCrAss001 virions created visible spots, but the intensity of the signal of most particles was too low to be detected by the software, indicating that the particle signal is just below the detection threshold of the device. Given that the size of the P1, T5 and ΦCrAss001 capsid is 90 nm or slightly below (Table 2), this suggests that the Videodrop detection threshold is slightly over 90 nm for phages.

In conclusion, the NanoSight and ZetaView correctly enumerated phage particles larger than 50 nm and 60 nm, respectively, consistent with previous studies on synthetic nanoparticles (Bachurski et al., 2019; Dehghani et al., 2021). The Videodrop detected and provided correct concentrations only for T4, a tailed phage with a capsid larger than 90 nm.

### 3.3 Particle size distributions of phage samples

In addition to the determination of particle concentrations, the three devices provide particle size distributions. The NanoSight provided size distributions in agreement with the samples’ characteristics, that is, in most cases, narrow unimodal distributions with a maximal value close to the diameter of the virions (Figure 3c and Table 3). For the tailless phages (ΦcrAss001, T7 and PRD1), the measured sizes were very close to the geometric diameters determined by microscopy (Tables 2 and 3 and Figure 3), indicating that the virions are close to perfect spheres. For phages with a large tail, we generally observed a bimodal distribution of sizes, with a first small peak corresponding to the geometric diameter of the capsid, hence to damaged tailless virions, and a second higher peak at higher particle size, most likely corresponding to intact virions, as the presence of a tail is expected to increase the hydrodynamic diameter of the virions (Figure 3). The bimodal distribution was particularly apparent in the case of the SPP1 sample, which was shown by TEM to contain close to 50% tailless capsids (Figure 1).

The ZetaView measured correct modal sizes for T4, P1, T5 and ΦcrAss001, but with poor precision, as evidenced by an important span of the size distributions (Table 3 and Figure 3). Similarly, the Videodrop measured a correct modal size for T4 and P1 (133 nm as compared to the geometric diameter of 120 nm for T4), also with poor precision (Figure 3c). In the case of phages whose diameter is close to the detection threshold (*λ*, T7 and PRD1), the ZetaView provided particle size distributions that were quite different from the expected distributions in terms of both mode and precision. In conclusion, the NanoSight provided more precise phage size estimations than the other devices, especially on smaller phages. It should be noted however that the size distributions provided by default by the NanoSight correspond to the measured sizes after transformation by the FTLA (Fine Track Lenght Adjustment) method, by opposition with the size distributions provided by the two other instruments, that correspond to raw measured diameters. The size distributions obtained by the three instruments should therefore only be compared with caution.

### 3.4 Quantification and size distribution of EVs of different origins

**FIGURE 4:**
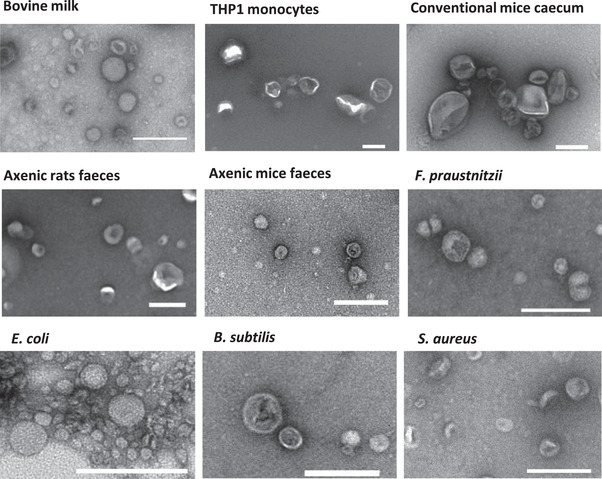
Representative TEM images of the EV preparations used in the study. Scale bars are 200 nm.

We next compared the performance of the three instruments in measuring the concentrations and size distributions of EV preparations from various origins. To this aim, we first prepared EV‐enriched preparations (called EVs from here on for simplicity) from nine different origins: bovine milk, THP1 human monocytic cell cultures, *F. prausnitzii*, *B. subtilis, E. coli* and *S. aureus* bacterial cultures, and, finally, EVs from germ‐free or conventional rodent faeces or caecal content. In germ‐free animals, EVs can exclusively originate from intestinal cells, whereas in conventional animals they also originate from the intestinal microbiota. Observations of EV preparations by TEM confirmed that most of the nanoparticles of the preparations were EVs, with a large dispersion of particle sizes (Figure 4 and Figure S3).

**FIGURE 5:**
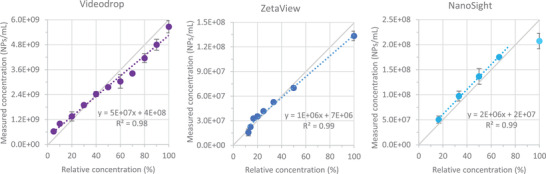
Concentration measurements of serial dilutions of EVs from bovine milk. Successive dilutions were realized in PBS. Error bars represent standard deviation on three independent dilutions.

**FIGURE 6:**
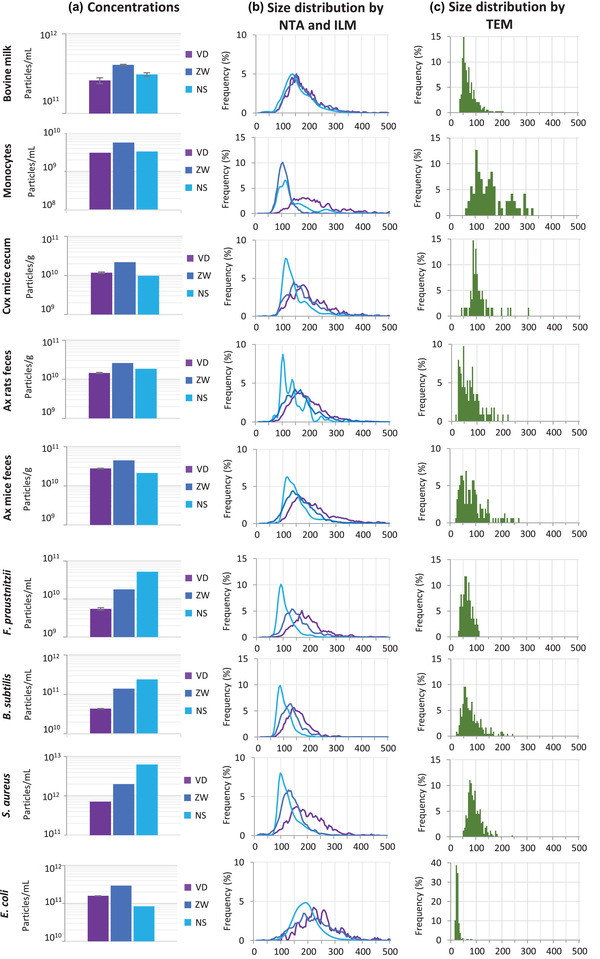
Concentration and sizes of EVs obtained with the different instruments. (A) Mean concentrations determined by the Videodrop (purple), the ZetaView (dark blue) and the NanoSight (clear blue). (B) Size distributions obtained with the three devices. Colors are as in (A). (C) Size distributions of particles in TEM images. Ax: axenic, Cvx: Conventional. Note that the y axis is different in B and C.

The EV preparations were then examined using the Videodrop, ZetaView, and NanoSight. As optimal concentration ranges vary with the nature of the sample and, in particular, with the level of polydispersity, we first determined the nanoparticle concentrations of serial dilutions of EVs from bovine milk, which range from 35 to 200 nm (Figures 5 and 6). For the NanoSight, the linearity ranges was much narrower than previously determined with polystyrene beads: they ranged from 2.5 × 10^7^ to only 2 × 10^8^ particles/mL instead of 1 × 10^7^ to 1 × 10^9^ particles/mL. This probably reflects the interference of large particles on the detection of small ones at high concentrations. On the contrary, the optimal concentration ranges for the Videodrop and the ZetaView were similar to that determined previously with polystyrene beads.

All EV preparations were then examined with the three optical devices. Contrary to phage samples, the concentrations of EVs were unknown and, therefore, we could only compare the values obtained with the three instruments to each other. Concerning EVs originating at least partially from mammalian cells (i.e., EVs from milk, THP1 monocytes or mouse intestinal contents), the concentrations determined by the three devices were relatively close, within a two‐fold range, the highest concentrations always being provided by the ZetaView (Figure 6a). This is unexpected as TEM suggested the presence, especially in samples from axenic animals, of EVs below the Videodrop detection threshold (90 nm) but larger than those of the NTA devices (60–70 nm). For example, in axenic mice samples, most EVs were comprised between 50 nm and 100 nm in size (Figure 6c). Of note, the EVs size detection thresholds were not determined here, however, given that the refractive index of EVs is only slightly lower than that of phages, the size detection thresholds should be similar for both types of objects. This would be in agreement with previous studies on EVs, in which the lower limit of detection of NTA was estimated to be 60–70 nm (Bachurski et al., 2019). A 10%–30% shrinkage of EVs is possible during sample preparation for TEM, which could explain part of this unexpected result. This could also be related to a hydrodynamic diameter of EVs larger that their geometric diameter, as already reported (see the Discussion).

By contrast, the devices were discordant in quantifying EVs originating from bacterial cultures, (except for *E. coli*, see below), with up to 10‐fold higher concentrations obtained with the NanoSight than with the Videodrop (Figure 6b). The sizes and levels of dispersion observed by TEM being similar in some eukaryotic and prokaryotic EVs (for example comparing the size distribution of EVs from axenic mice faeces and from *B. subtilis*), the different outcome could be the result of dissimilar characteristics of bacterial and mammalian EVs. The case of *E. coli* EVs is particular, as TEM revealed that most EVs had a diameter between 20 and 40 nm, in agreement with previous publications (Fabrega et al., 2017; Perez‐Cruz et al., 2016), and therefore are much smaller than the detection thresholds of the three devices. In this case, the concentrations measured were therefore probably strongly underestimated with all three devices.

Concerning EV size distributions, all devices provided size distributions that were both shifted toward larger values and broader than the geometrical diameters obtained by TEM (Figure 6). This trend is more pronounced for the Videodrop and ZetaView than for the NanoSight. This suggest that, contrarily to phage virions, EVs are far from being perfect spheres (they are soft and charged) and that hydrodynamic diameters are quite different from geometrical ones.

### 3.5 Comparison of data acquisition and analysis times with the three devices

**FIGURE 7:**
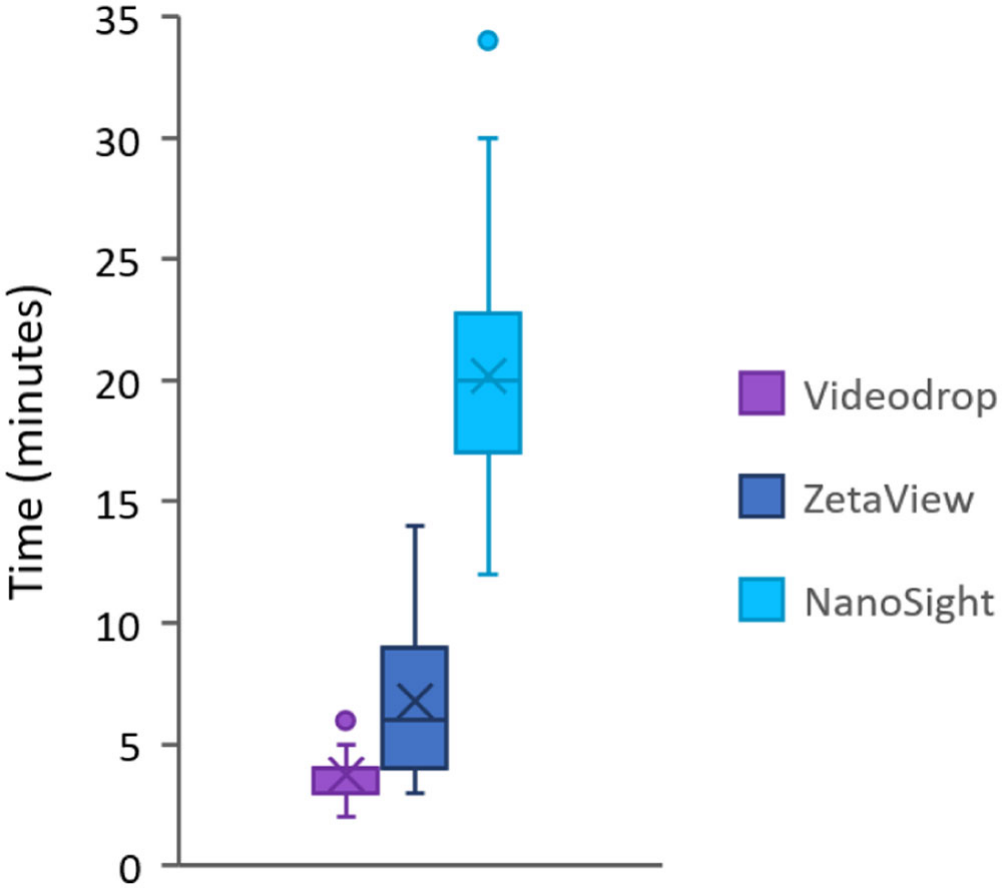
Distribution of the total time of sample analysis. This time includes sample positioning, acquisition of movies and analysis.

Finally, to objectify the greater speed of analysis with the Videodrop than with the two NTA devices, we evaluated the time necessary for each observation with the three devices by using the experiment file saving time. The mean acquisition time was 3.7, 6.7 and 20.2 min with the Videodrop, the ZetaView and the NanoSight, respectively (Figure 7). This difference in the time of sample analysis is mostly related to three aspects: (i) the positioning of the samples, (ii) manual user calibration steps required with the NanoSight (as compared to automatic calibration with the Videodrop), and (iii) the number of nanoparticles recorded per experiment (though this last point can be modified by the user for the three devices). In addition, the variance is much higher with the NanoSight, reflecting that problems regularly arise during the acquisition, such as the presence of air bubbles trapped in the acquisition chamber that need to be dislodged.

## 4 DISCUSSION

As a first step towards evaluation of the Videodrop, we precisely determined the concentration of phages using three classical methods. We established that qPCR and EPI provide very similar concentrations, in contrast to a previous study in which the authors found significantly lower concentrations by EPI than qPCR (Kaletta et al., 2020). The better agreement between the values obtained by the two methods in our study may have resulted from the use of a more concentrated staining solution, the addition of a flash freezing step to improve the staining, and/or the use of a different microscopic setup. With these phage preparations whose concentration has been rigorously established, we found that only the large T4 phage (86 × 120 nm) could be precisely enumerated by the Videodrop. A linear count consistent with the dilution was observed for concentrations between 5 × 10^8^ and 4 × 10^9^ particles/Ml, indicating the successful enumeration of individual virions. About 80–90‐nm diameter virions of P1, T5 and ΦcrAss001 were detected by the Videodrop, but their concentrations were underestimated by 3–10‐fold. This suggests that the size threshold of this instrument for phage enumeration is approximately 90 nm. This contrasts with the reported good performance of the Videodrop for the quantification of an adenovirus of 75.5 nm of diameter: in this study, the authors could detect approximately 25% of adenovirus particles Two factors could explain this difference. First, the refractive index of adenoviruses or their surface charge may be higher than that of the phages tested here, improving their detection. Second, the adenovirus observations were carried out using a relative threshold of 3.2, whereas we used a relative threshold of 3.8, consistent with the manufacturer's recommendations, to limit the number of fake positive detections. Concerning the two NTA instruments, our results suggest a sensitivity threshold for phages < 60 nm of diameter for NanoSight and between 60 and 75 nm for Zetaview under the selected experimental conditions, which is in accordance with previous results on EVs (van der Pol et al., 2014).

In terms of the determination of nanoparticle size distributions, the NanoSight provided very precise and accurate measurements of capsid sizes for tailless phages, indicating that phage hydrodynamic diameters are close to their geometric diameters. In polydisperse EV samples, however, we show that ILM results in a broader and shifted distribution of EV sizes, similar to what others have already observed for NTA (Bachurski et al., 2019). This difference is too great to be solely explained by the shrinking phenomenon sometimes observed with TEM (Chernyshev et al., 2015; Kotrbova et al., 2019). Instead, this overestimation most likely results from several phenomena inherent to NTA. First and probably most importantly, EVs smaller than the detection threshold of the devices (60–70 nm for NTA and 90 nm for ILM) are not detected by NTA. In addition, small particles close to the detection limit are difficult to track, due to low intensity and rapid movement, and therefore often cannot be attributed a size. Third, the protein surface cargo influences the hydrodynamic diameter measured by NTA (Bachurski et al., 2019; Skliar et al. 2018), resulting in sizes measured by NTA larger than the geometric diameters. Finally, in NTA‐based devices, large particles mask small ones, as the detected signal is proportional to the sixth power of the particle's diameter an object, which makes an object 10 times bigger than another diffuse a million times more light (Dehghani et al., 2021; Ortega Arroyo et al., 2016). In theory, ILM is less subject to such a masking effect because the detected signal is proportional to the third power of the diameter of the nanoparticles. This could explain why, despite the lower sensitivity of the Videodrop instrument, it was able to determine EV concentrations similar to those of the NTA instruments in milk and mouse intestinal samples, which are highly polydisperse in size.

In any case, size measurements provided by NTA and ILM instruments must be considered with caution, as they do not reflect the true geometrical size distribution. The case of *E. coli* EVs particularly highlights the importance of coupling TEM observations with ILM or NTA. Indeed, in this sample, TEM showed that most vesicles were <50 nm in diameter, and therefore smaller than the detection threshold of ILM and NTA. Thus, the concentrations obtained with these devices were clearly underestimations.

On another level, a major advantage of the Videodrop instrument is its ease and speed of use: in a 3‐h work period, as much as 50 samples can be analysed with the Videodrop, as compared to only about ten with the NanoSight (Table 4). This is mostly related to the very rapid sample positioning in the Videodrop (a 6‐μL drop of the sample has to be deposited into a dedicated microcavity) relative to positioning of the sample through a syringe in NTA, which has to be very carefully connected to the tubing to avoid generating air bubbles and necessitate to wait for the stabilization of the flux. In addition, the time of acquisition of movies and data analysis is much longer with the NanoSight. The ZetaView presents similar limitations as the NanoSight; however, although it necessitates an initial calibration step prior use, this device offers better efficiency in terms of the time of analysis. Another advantage of the sample positioning of the Videodrop is that it enables the measurement of samples rich in diverse contaminants, such as soluble lipids and proteins, which is not possible with the NTA devices, as such contaminants could result in clogging of the tubing. For example, in the context of this study, raw faecal filtrates could not be observed with the NTA instruments, as they contain flagella, lipids, and proteins that are either soluble or in aggregates, whereas such raw filtrates could be observed with the Videodrop.

**TABLE 4:** Evaluation summary of the Videodrop, ZetaView and NanoSight.

	**Videodrop**	**ZetaView**	**NanoSight**
Calibration of the device before use	No	Yes (5‐10 min)	No
Manual determination of acquisition parameters	No	Yes	Yes
Tolerates the presence of lipidic or proteic contaminants in samples	Yes	No	No
Tolerates the presence of detergents in samples	Yes	No	No
Optimal concentration range for EV measurments (part/mL)	5.0x10^8^‐4.0x10^9^	2.5x10^7^‐2.0x10^8^	2.5x10^7^‐2.0x10^8^
Number of tracked particles per acquisition under selected experimental conditions	200‐1,000	1,000‐1,500	1,000‐10,000
Phage size detection threshold for absolute counting and sizing	Between 90 and 100 nm	Between 60 and 90 nm	Between 30 and 60 nm
Number of samples that can be analysed in 3 h	∼50	15‐20	5‐10

A final advantage of the Videodrop is the automatic adjustment of the acquisition parameter for the video capture, here LED intensity. By contrast, NTA requires several optimization steps to determine the suitable settings for video capture and analysis. As reported previously (Bachurski et al., 2019; Maas et al., 2015), the camera settings in both devices (NanoSight NS300: camera level, ZetaView: camera sensitivity) have a profound impact on the measured concentration. In addition, the optimal settings are very difficult, if not impossible, to determine for polydisperse samples, as raising the level of the camera results in the better observation of small particles but also increased noise created by large ones (and, conversely, lowering the level of the camera is better for large‐particle enumeration but leads to the loss of small particles).

In conclusion, the better sensitivity of the NanoSight instrument makes it the most appropriate device for concentration determinations of monodisperse populations of objects between 50 and 70 nm in diameter. In addition, of the three devices we tested, the NanoSight provided the most precise size distributions and projects requiring precision on a limited number of samples would benefit the most from this device. However, the higher precision and sensitivity of the NanoSight is offset by four disadvantages: its time‐consuming manipulation, its high susceptibility to masking effects, the impossibility to analyse samples that contain a large number of impurities, and its price. Depending on the application, the most important advantage of the Videodrop would be its ease of use and the rapidity of sample observation (Table 2). In addition, ILM is less prone to small‐particle masking by larger particles and thus compares favourably with NTA for concentration measurements of highly polydisperse populations. Finally, the Videodrop is less expensive and requires fewer consumables than the other devices (a pipet tip as opposed to a syringe), which is directly related to a lower environmental footprint, which should also be taken into consideration.

## AUTHOR CONTRIBUTIONS

**Romain Sausset**: Conceptualization; Data curation; Formal analysis; Investigation; Methodology; Resources; Validation; Visualization; Writing—original draft; Writing—review & editing. **Zuzana Krupova**: Conceptualization; Investigation; Methodology; Resources; Writing—review & editing. **Eric Guédon**: Resources; Writing—review & editing. **Sandrine Peron**: Investigation; Resources. **Marie‐Agnès Petit**: Conceptualization; Supervision; Writing—review & editing; Writing—original draft. **Alice Grangier**: Investigation; Resources; Writing—review & editing. Luisa De Sordi: Conceptualization; Funding acquisition; Methodology; Supervision; Writing—original draft; Writing—review & editing. **Marianne De Paepe**: Conceptualization; Data curation; Formal analysis; Funding acquisition; Investigation; Methodology; Project administration; Supervision; Validation; Visualization; Writing—original draft; Writing—review & editing.

## CONFLICTS OF INTEREST STATEMENT

The funders had no role in the design, analysis or interpretation of the results.

## Supporting information

Supplementary Information
