# Supplementary material for: Comparison of interferometric light microscopy with nanoparticle tracking analysis for the study of extracellular vesicles and bacteriophages
Source: J Extracell Biol. 2023 Feb 23;2(2):e75. doi: 10.1002/jex2.75 (PMC11080698; doi:10.1002/jex2.75)
Supplement: Supplementary file 1 — Supplementary Information [file JEX2-2-e75-s001.pptx]

## Slide 1
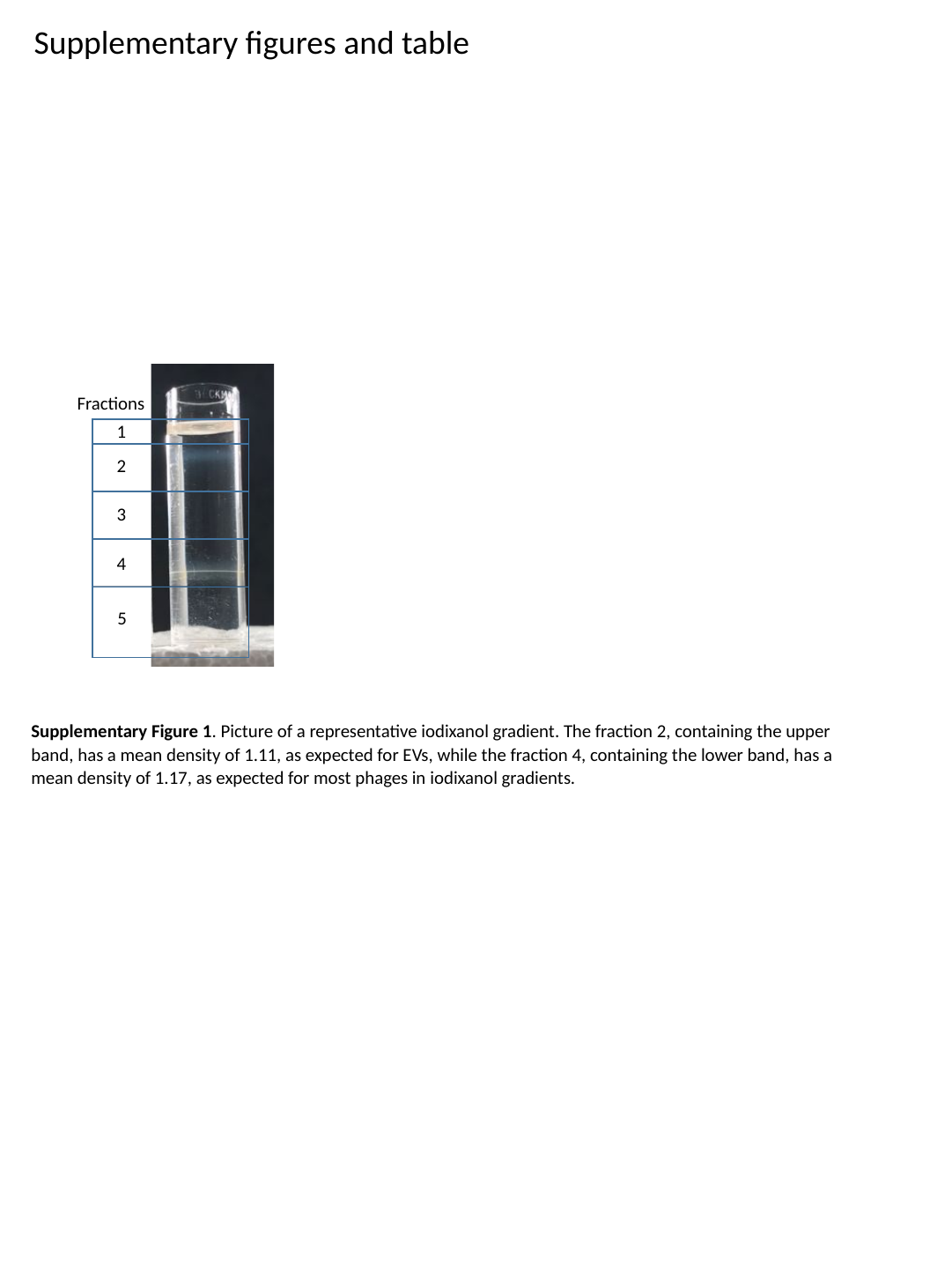

Supplementary figures and table
Fractions
1
2
3
4
5
Supplementary Figure 1. Picture of a representative iodixanol gradient. The fraction 2, containing the upper band, has a mean density of 1.11, as expected for EVs, while the fraction 4, containing the lower band, has a mean density of 1.17, as expected for most phages in iodixanol gradients.

## Slide 2
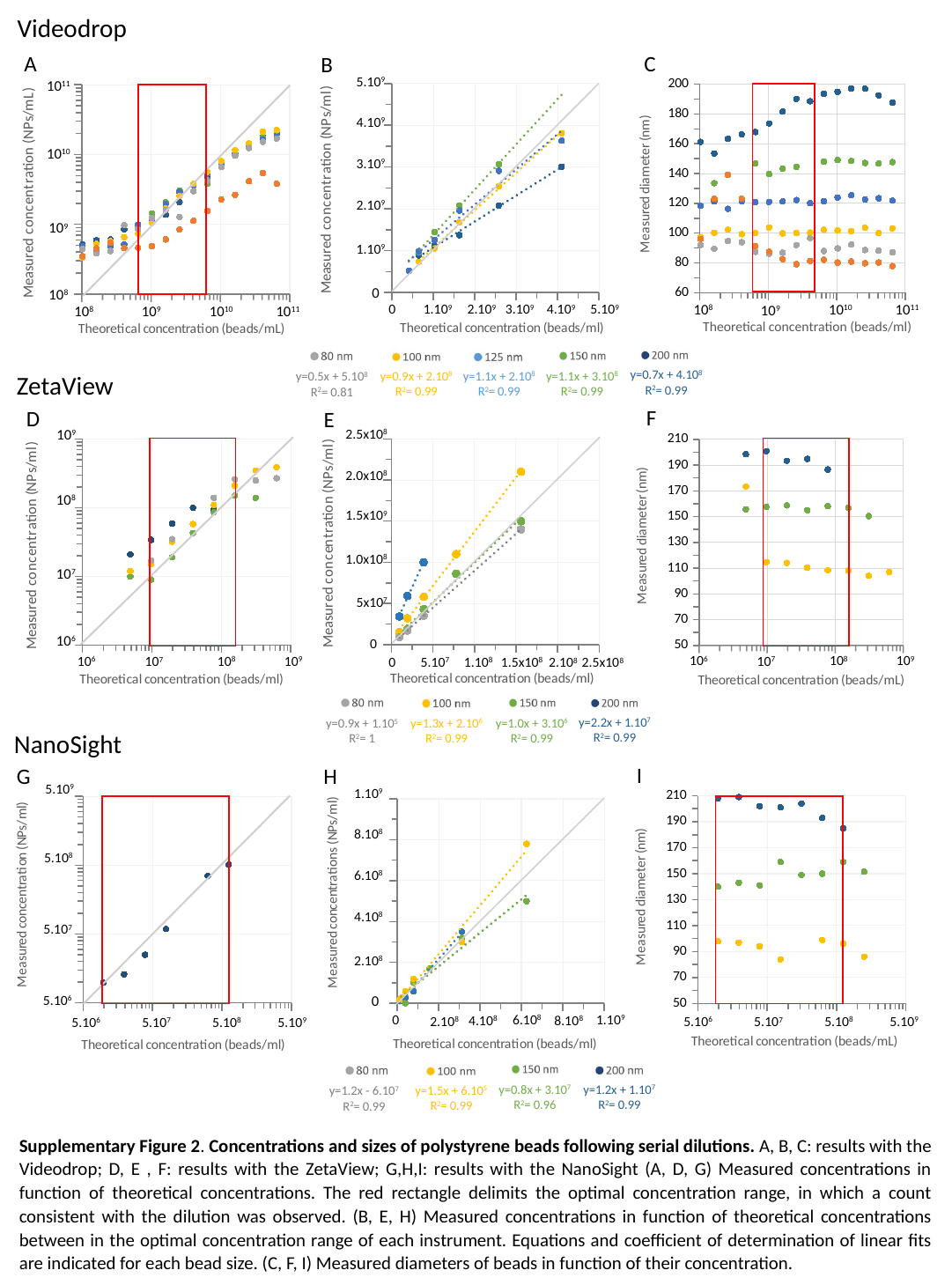

Videodrop
C
A
B
5.109
### Chart
| Category | 80 nm | 100 nm | 125 nm | 150 nm | 200 nm |
|---|---|---|---|---|---|
### Chart
| Category | 70 nm | 80 nm | 100 nm | 125 nm | 150 nm | 200 nm |
|---|---|---|---|---|---|---|1011
### Chart
| Category | 70 nm | 80 nm | 100 nm | 125 nm | 150 nm | 200 nm |
|---|---|---|---|---|---|---|
4.109
1010
3.109
2.109
109
1.109
0
108
108
1010
1011
0
1.109
2.109
3.109
4.109
5.109
109
108
1010
1011
109
Theoretical concentration (beads/ml)
y=0.7x + 4.108
R2= 0.99
y=1.1x + 3.108
R2= 0.99
y=0.5x + 5.108
R2= 0.81
y=0.9x + 2.108
R2= 0.99
y=1.1x + 2.108
R2= 0.99
ZetaView
F
D
E
109
2.5x10^8^
### Chart
| Category | 80 nm | 100 | 150 | 200 |
|---|---|---|---|---|
### Chart
| Category | 80 nm | 100 | 150 | 200 |
|---|---|---|---|---|
### Chart
| Category | 80 nm | 100 | 150 | 200 |
|---|---|---|---|---|
2.0x10^8^
108
1.5x10^9^
1.0x10^8^
107
5x10^7^
106
0
106
108
109
107
106
108
109
107
5.107
1.108
1.5x10^8^
0
2.108
2.5x10^8^
y=1.0x + 3.106
R2= 0.99
y=0.9x + 1.105
R2= 1
y=1.3x + 2.106
R2= 0.99
y=2.2x + 1.107
R2= 0.99
NanoSight
I
G
H
### Chart
| Category | 80 nm | 100 | 150 | 200 |
|---|---|---|---|---|5.109
### Chart
| Category | 80 nm | 100 | 150 | 200 |
|---|---|---|---|---|1.109
### Chart
| Category | 80 nm | 100 | 150 | 200 |
|---|---|---|---|---|
8.108
5.108
6.108
4.108
5.107
2.108
5.106
0
1.109
6.108
0
4.108
8.108
5.106
5.108
5.109
2.108
5.107
5.106
5.108
5.109
5.107
y=0.8x + 3.107
R2= 0.96
y=1.2x - 6.107
R2= 0.99
y=1.5x + 6.105
R2= 0.99
y=1.2x + 1.107
R2= 0.99
Supplementary Figure 2. Concentrations and sizes of polystyrene beads following serial dilutions. A, B, C: results with the Videodrop; D, E , F: results with the ZetaView; G,H,I: results with the NanoSight (A, D, G) Measured concentrations in function of theoretical concentrations. The red rectangle delimits the optimal concentration range, in which a count consistent with the dilution was observed. (B, E, H) Measured concentrations in function of theoretical concentrations between in the optimal concentration range of each instrument. Equations and coefficient of determination of linear fits are indicated for each bead size. (C, F, I) Measured diameters of beads in function of their concentration.

## Slide 3
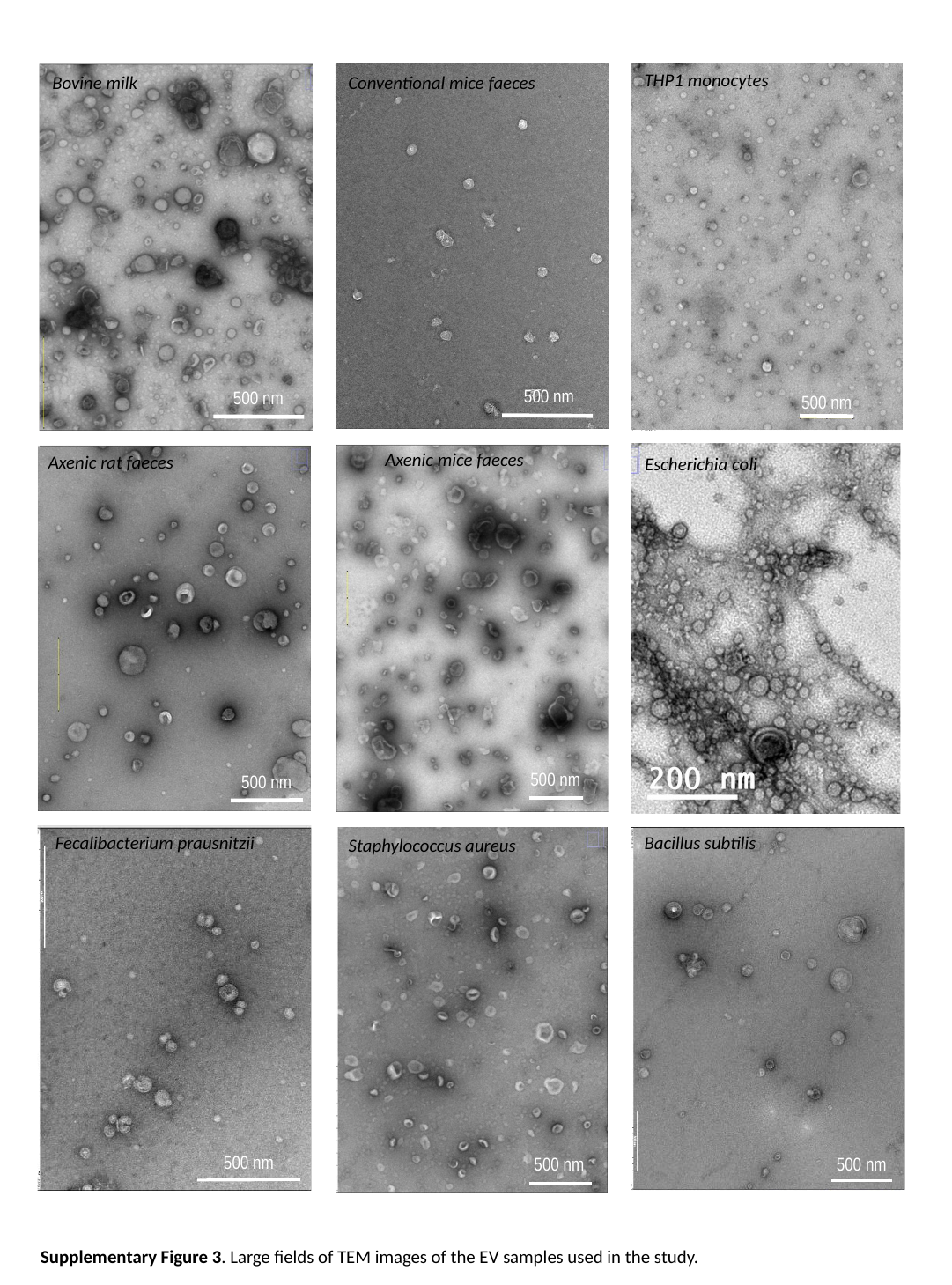

Thp1 monocytes
Bovine milk
Conventional mice faeces
1 µm
Axenic mice faeces
500 nm
500 nm
500 nm
Axenic mice faeces
Axenic rat faeces
Escherichia coli
500 nm
500 nm
Bacillus subtilis
Staphylococcus aureus
Fecalibacterium prausnitzii
500 nm
500 nm
500 nm
Supplementary Figure 3. Large fields of TEM images of the EV samples used in the study.

## Slide 4
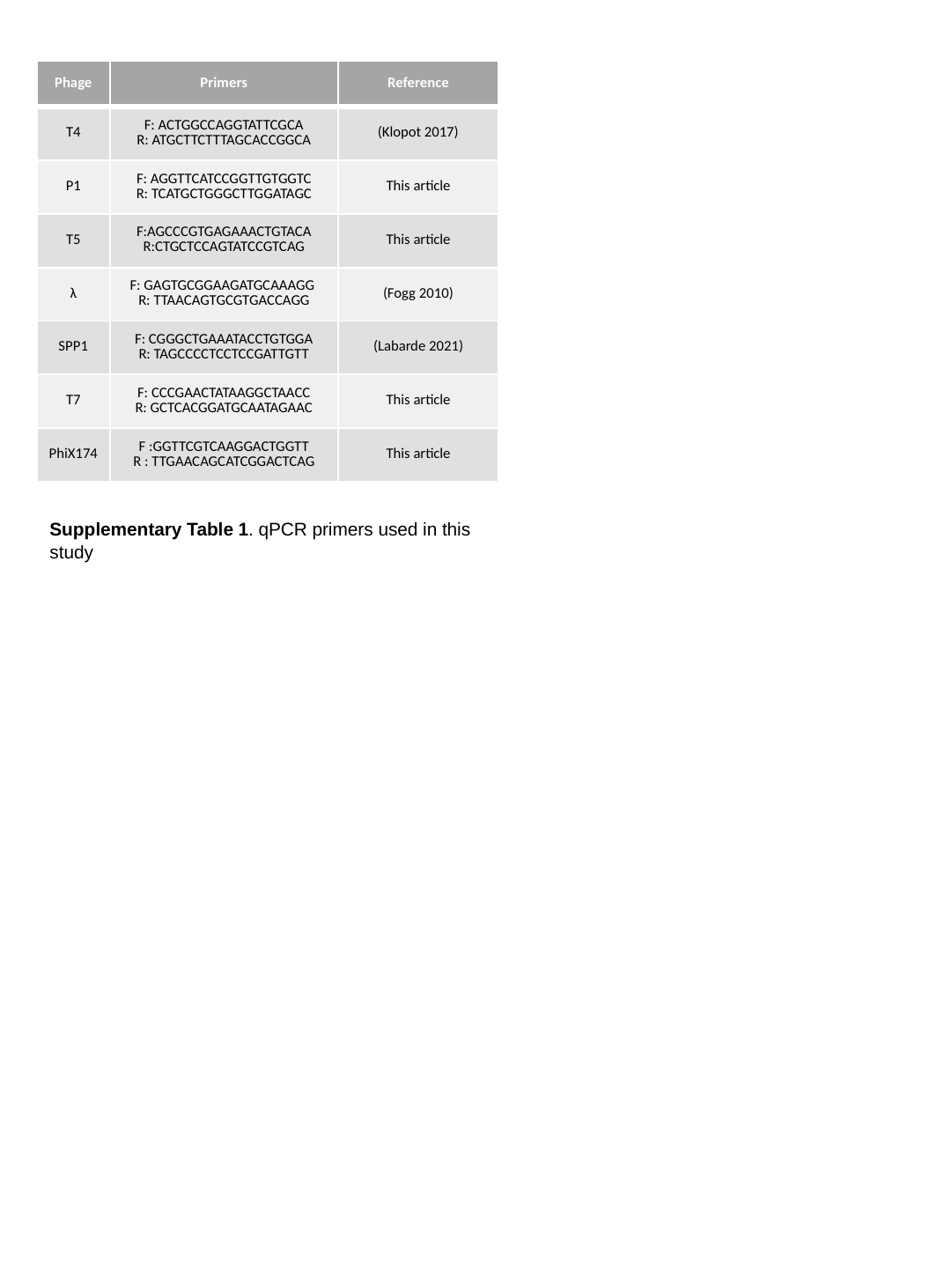

| Phage | Primers | Reference |
| --- | --- | --- |
| T4 | F: ACTGGCCAGGTATTCGCA R: ATGCTTCTTTAGCACCGGCA | (Klopot 2017) |
| P1 | F: AGGTTCATCCGGTTGTGGTC R: TCATGCTGGGCTTGGATAGC | This article |
| T5 | F:AGCCCGTGAGAAACTGTACA R:CTGCTCCAGTATCCGTCAG | This article |
| λ | F: GAGTGCGGAAGATGCAAAGG R: TTAACAGTGCGTGACCAGG | (Fogg 2010) |
| SPP1 | F: CGGGCTGAAATACCTGTGGA R: TAGCCCCTCCTCCGATTGTT | (Labarde 2021) |
| T7 | F: CCCGAACTATAAGGCTAACC R: GCTCACGGATGCAATAGAAC | This article |
| PhiX174 | F :GGTTCGTCAAGGACTGGTT R : TTGAACAGCATCGGACTCAG | This article |
Supplementary Table 1. qPCR primers used in this study
